# Predicting Physical Activity Behavior by Automatic and Reflective Self-Regulatory Processes

**DOI:** 10.3389/fpsyg.2021.714608

**Published:** 2021-10-21

**Authors:** Ines Pfeffer, Tilo Strobach

**Affiliations:** ^1^Department of Pedagogy, Faculty of Human Sciences, Medical School Hamburg, Hamburg, Germany; ^2^Department of Psychology, Faculty of Human Sciences, Medical School Hamburg, Hamburg, Germany

**Keywords:** exercise, automatic associations, implicit association test, executive functions, shifting, inhibition, updating

## Abstract

In this study, we examined the interaction of automatic (i.e., automatic affective evaluations) and reflective [i.e., reflective intention and executive functions (EFs)] processes on physical activity (PA) behavior based on dual-process theories. We expected main effects as well as significant interaction effects between automatic associations, intention, and EFs on behavior. In particular, a well-controlled implicit-association-test (IAT) was applied to assess automatic affective evaluation. A prospective study with two points of measurement (*N*=212 students) was conducted. At t1, age, sex, PA behavior (control variables), automatic associations, EFs (shifting, updating, inhibition), and PA intention (predictors and moderators) were assessed with standardized questionnaires and tests. At t2 (4weeks later), PA behavior (dependent variable) was measured with a standardized questionnaire. A hierarchical multiple linear regression analysis including two- and three-way interactions between IAT results, intention, and EFs on PA behavior was conducted. Results showed that the interactions Intention x Shifting and IAT x Intention x Inhibition were significant. Moderation analyses revealed that participants with higher intentions and lower inhibition values (improved inhibition abilities) showed a negative association between IAT and PA, while those with lower intentions and lower inhibition values showed a positive association between IAT and PA, which was documented in a significant slope difference test between these two groups. Thus, both automatic and reflective processes contribute and interact in predicting PA. As well as fostering more positive affective evaluations towards PA, interventions to strengthen PA intentions and to improve EFs could help to increase PA behavior.

## Introduction

Theories assume that health behavior regulation is basically related to two types of information processing: automatic processes (i.e., autonomous; independent of working memory) and reflective processes (i.e., controlled; requiring working memory; Strack and Deutsch, 2004; Hofmann et al., 2009; De Houwer and Moors, 2012; Brand and Ekkekakis, 2018; Melnikoff and Bargh, 2018). However, the specific characteristics and mechanisms of how automatic and reflective processing work together in guiding PA behavior are still understudied. Therefore, it is aimed to examine the relationship between theory-driven, well-established constructs, and to integrate them in a dual-process model of PA self-regulation (Strobach et al., 2020). In particular, it is examined how affective evaluations, representing a relevant aspect of automatic processing, as well as PA intention (i.e., the expression of motivation) and executive functions (EFs; as control mechanisms of human actions and a core component of self-regulation abilities), as relevant aspects of reflective processing, are associated with PA behavior. By doing so, we explain how automatic and reflective processes regulate, control, and coordinate such behavior.

### Dual-Process Theories

General dual-process theories of human behavior (Strack and Deutsch, 2004; Hofmann et al., 2009) and dual-process theories on PA (Brand and Ekkekakis, 2018; Strobach et al., 2020) suggest that this behavior is controlled by two types, automatic and reflective processes, which operate in parallel, but may also interact in regulating behavior (Muschalik et al., 2018). Automatic processes require only few, if any working memory resources, and control behavior in suboptimal conditions (e.g., when self-control is low). Contrastingly, reflective processes model behavior through reflective decision making, such as balancing behavior costs and benefits or the exertion of self-control to overcome barriers in an effortful mode. These reflective processes mainly require working memory capacities, therefore making them susceptible for interference by conflicting and distracting information, which might cause these processes to fail (Arnautovska et al., 2017). Importantly, the availability of self-control abilities determines whether automatic or reflective processing is the dominating type to behaviour control in a particular situation (Perugini et al., 2010; Brand and Ekkekakis, 2018).

### Automatic Processes

In the context of PA behavior two prominent concepts have been used to examine PA automatic processes: (1) habits (i.e., behavioral automaticity) and (2) automatic affective evaluations; these concepts should not be used equivalently, even though they might share some common variation (De Houwer and Moors, 2012; Hagger, 2020). The concept of habits assumes that behavior is largely initiated and executed automatically (with low reflective processing) in a specific context. However, according to Hagger (2020) habits seem to be aligned with other automatic processes that determine behavior and should be seen as a part of an overarching set of automatic processes. On the other hand, automatic affective evaluations reflect the affective experiences that arise rapidly and involuntarily when the concept of PA is activated in a person’s mind (Conroy and Berry, 2017; Brand and Ekkekakis, 2018). It is possible that habits are a function of a behavioral schema and that the habitual behavior could be initiated through activation of different types of information held on the schema, either by cue/context-response pairings or by activation of automatic affective evaluations (Hagger, 2020).

Recent research has demonstrated that automatic affective evaluation moderated the relationship between self-reported habits and behavioral enactment (Phipps et al., 2021); the effect of habits on behavior was largest when automatic affective evaluations were positive. Investigating the predicting impact of self-reported habit on PA in the context of dual-process theories, a recent study empirically revealed that habit was directly associated with PA behavior and additionally qualified in an interaction. In this interaction of automaticity, intention, and trait self-control, automaticity had the strongest association with PA behavior when intention and trait self-control were lower compared to higher (Pfeffer and Strobach, 2020b), i.e., automaticity guided PA behavior in general and in particular in individuals with weaker intention and lower trait self-control.

In another study, individuals with higher PA levels, compared with individuals with lower levels, showed stronger exercise–approach versus exercise–avoid memory associations (Hannan et al., 2019). Moreover, an approach bias for PA cues explained unique variance in self-reported PA after controlling for explicit PA intentions and self-determined PA motivation, indicating that both automatic and reflective processes were simultaneously associated with behavior. In the study of Padin et al. (2017) automatic affective evaluations and trait self-regulation were not significantly related to PA behavior. However, there was a significant interaction of automatic affective evaluations of PA and trait self-regulation. Automatic evaluations were unrelated to average workout length when trait self-regulation was higher. However, individuals with more negative automatic evaluations and lower trait self-regulation demonstrated shorter workout sessions compared to those individuals with more positive attitudes. This finding indicated that lower self-regulation and more negative automatic affective evaluations may impede PA behavior enactment.

Generally, previous results indicate that habits and automatic affective evaluations directly and interactively predict PA. However, the self-report measurement of habit as a cue–response relationship is susceptible to response biases (e.g., by social desirability and self-presentational concerns) and habits are hardly introspectively accessible using self-report questionnaires as conducted in Pfeffer and Strobach (2020b; see also Rebar et al., 2018). As an alternative to introspective accessibility, measures, such as response latencies, should be best able to reflect the strength of individuals’ automatic processing. Consistent with the assumptions of the Affective-Reflective Theory of physical inactivity and exercise (Brand and Ekkekakis, 2018), it is assumed that automatic associations towards a behavior awake an affective evaluation that activates an approach or avoidance tendency (Conroy and Berry, 2017). This tendency is conceptualized as the default response to which the slower reflective processes are added to (i.e., the latter processes work in addition to automatic processes). In contrast to habits, such implicit measures rather assess automatic affective evaluations between, for example, cue and behavior (Hagger et al., 2015).

Due to the difficulties in assessing automatic processes such as habits with introspection, it is relevant to investigate automatic affective evaluations as a core component of automatic processes in a standardized and computerized test. As a solution for this investigation, the Implicit Association Test (IAT) provides a measure of strengths of automatic affective evaluation (Greenwald et al., 2002; Nosek et al., 2007; Rebar et al., 2018). The apparent usefulness of the IAT may also be due to its combination of apparent resistance to self-presentation artifact (Kim and Greenwald, 1998; Banse et al., 2001; Egloff and Schmukle, 2002) and its ease of adaptation to assess a broad variety of socially significant automatic affective evaluations (see overview in; Greenwald and Nosek, 2001).

We assume that automatic processes are the default response to which the reflective processes are added to, and will guide behaviour in case that reflective processes are not available. However, they can be voluntarily overridden by instigating reflective processes. For example, a person that holds negative automatic affective evaluations towards PA might show an avoidance tendency to be physically active, which collides with their intention (a result of reflective processing) to be more physically active. In this case, the exertion of self-control is needed in order, for example, to inhibit the avoidance tendency and to translate the intention into PA behavior.

### Reflective Processes

Reflective social-cognitive models often explain PA by the reflective component intention (i.e., motivation for a specific behavior) as its most important and proximal predictor (Ajzen, 1991; Bandura, 1991; Rhodes and Dickau, 2013). Intention can be seen as a result of reflective processing, such as weighting up advantages and disadvantages of being physically active or considering self-efficacy experiences. However, individuals often disappoint in transferring their PA intentions into PA behavior. This intention–behavior gap (Rhodes and de Bruijn, 2013; Sheeran and Webb, 2016) can be overcome by self-regulation, i.e., by controlling one’s behavior, emotions, and thoughts in the pursuit of long-term goals and to overcome, e.g., potential barriers (e.g., bad weather, negative affective evaluations towards the behavior or lack of time when intended to go running).

Emerging evidence suggests that EFs play an important role in effective self-regulation of positive health behaviors and the intention–behavior gap. For example, EFs play an important role in overriding dominant automatic processes and associated behavioral tendencies that might impede goal attainment (Hall and Fong, 2007, 2013; Best et al., 2014; Buckley et al., 2014). EFs are those cognitive concepts that refer to goal-directed and higher level cognitive processing. They enable effortful top-down control of behavior over lower level cognitive processes. These functions’ combination is a multifaceted construct comprised of several higher level control processes that subserve the ability to self-regulate, wherein individual differences in these processes predict the transfer of intentions into actions (Hofmann et al., 2012). In their *unity/diversity framework*, Miyake and colleagues (Miyake et al., 2000; Miyake and Friedman, 2012) systematized the complexity of different situations and processes involving the EF construct primarily in three domains: *inhibition*, *updating*, and *shifting*. Inhibition is related to deliberate overriding of dominant or prepotent responses, updating refers to monitoring and manipulating working memory contents, and shifting is associated with switching flexibly between different tasks or mental sets (i.e., cognitive flexibility). The unity/diversity framework states that while the executive domains tap some common variability (i.e., the unity component), they are also separable (i.e., the diversity component). This common/separable variability is implicitly assessed by analyzing behavioral performance in EF tests (e.g., the task switching test to measure shifting).

One main component of successful self-regulation is the ability to actively inhibit or suppress inappropriate behavioral responses (i.e., automatic processes such as bad habits and inappropriate impulses), being incompatible with one’s goals and intentions (Hofmann et al., 2012). Empirical evidence has demonstrated that individuals with low levels of inhibition performance are less successful at translating their intention of health behavior into actual behavior (Hall et al., 2008; Allan et al., 2011). Further, Hall et al. (2008) have shown that performance levels in inhibition moderated the intention–behavior gap in PA. Individuals with improved inhibition performance demonstrated stronger associations between intention and PA than those with lower inhibition performance.

However, it is an open issue how the executive domains updating and shifting are associated with PA behavior and, in particular, with the gap between intention and PA behavior (Pfeffer and Strobach, 2017, 2020b). Pfeffer and Strobach (2017) demonstrated that performance in updating is associated with the PA intention–behavior gap and that performance in inhibition, updating, and shifting tests moderate the association between the trait self-control and this PA gap. We thus showed that the complex pattern, modulating the association between intended and realized PA behavior, includes trait self-control, EFs, as well as a combination of these self-regulation components. Several studies have shown that having a health-related goal (e.g., the intention to be physically active) may only be beneficial when an individual has sufficient updating ability (Hofmann et al., 2008, 2009; Allan et al., 2013; Allom and Mullan, 2014), and it is argued that updating might be more important for the self-regulation of PA behavior than inhibition (Pfeffer and Strobach, 2017, 2020a). Furthermore, it was shown that people with greater shifting abilities use more flexible self-regulation, leading to greater PA levels (Kelly and Updegraff, 2017). That is, although there is an integration of inhibition with the domains updating and shifting in the unity/diversity framework (e.g., Miyake et al., 2000), there are investigations needed that associate EF domains with this gap in a systematic and elaborative way. For instance, it can be postulated that inhibition performance interacts with affective evaluations and PA intention, so that lower inhibition performance and intention, compared to higher inhibition performance and intention, leads to a stronger impact of affective evaluations on PA, as automatic processes are thought to prevail, when reflective processes are impaired.

### The Present Study

While previous studies investigated the impact of PA affective evaluations, intentions, and EFs on PA behavior rather separately, they are lacking combined investigations in the framework of dual-process models. We assume that automatic processes are the default response to which the reflective processes are added to, and automatic will guide behavior in case that reflective processes are not available. However, automatic processes can be voluntarily overridden by instigating reflective processes. For example, a person that holds negative affective evaluations towards PA might show an avoidance tendency to be physically active, which collides with their intention (a result of reflective processing) to be more physically active. In this case, the exertion of self-control is needed in order, for example, to inhibit the negative evaluation and the avoidance tendency of PA and to still translate the PA intention into behavior. Therefore, individuals with rather negative evaluations of PA might also perform a high amount of PA when having strong intentions to be physically active and having higher EFs (i.e., better abilities to self-regulate).

**Figure 1:**
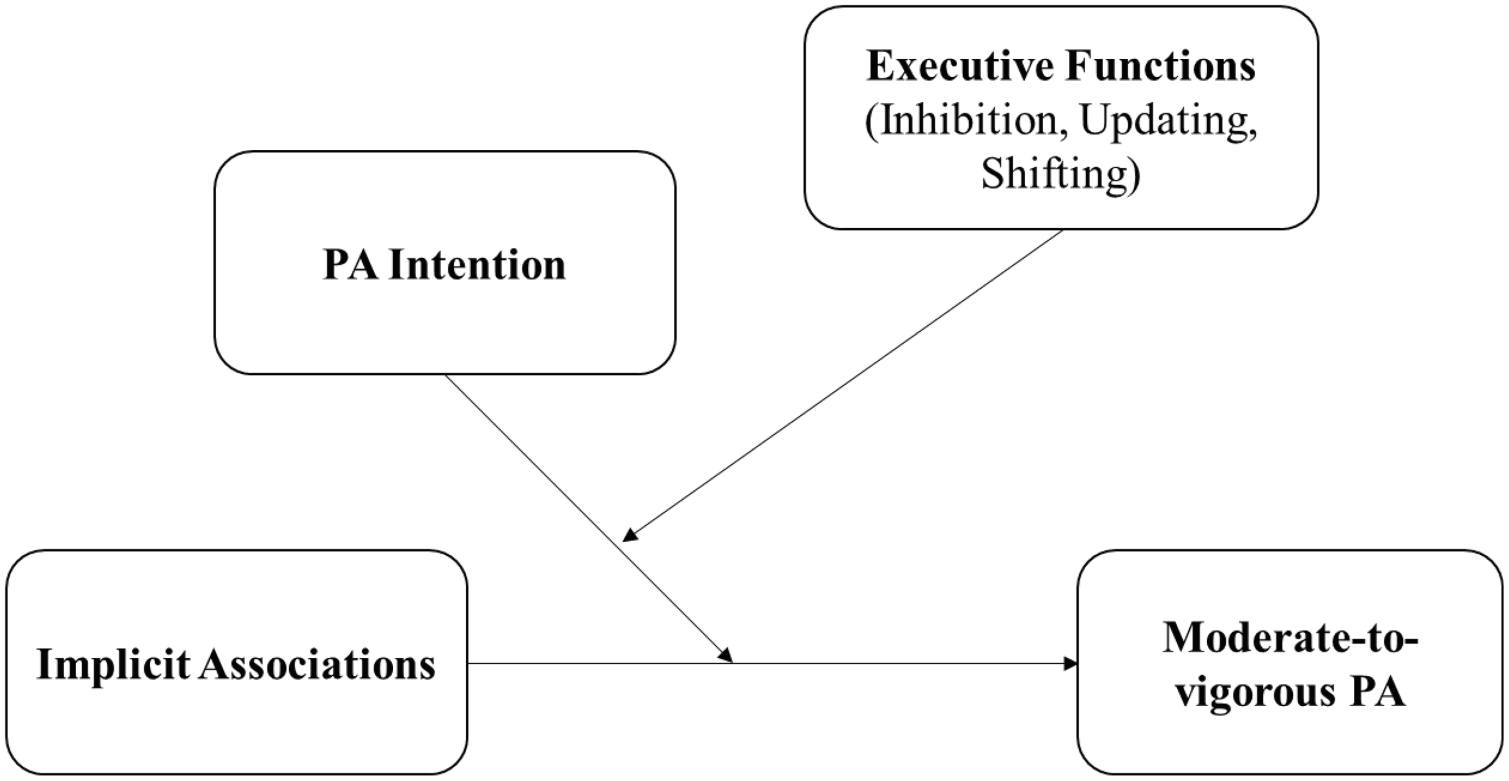
Conceptual research model based on dual-process theories. PA = physical activity.

Based on these assumptions, we aimed to examine the direct effects and interactions of automatic and reflective factors on PA in the context of dual-process theories. That is, we tested the direct effects and two-way as well as three-way interactions of PA affective evaluations, intentions, and EFs on PA within one statistical model. We hypothesize in line with the results of Pfeffer and Strobach (2020b) that (1) PA affective evaluations, intentions, and EFs are positive predictors of PA behavior and (2) PA affective evaluations and intention, PA affective evaluations and EFs as well as intention and EFs interact in predicting PA behavior. In detail, PA affective evaluations are a stronger predictor of behavior when (a) intention, and (b) EF performances are lower compared to higher. Furthermore, (c) intention is a stronger predictor of behavior when EF performances are higher compared to lower. We additionally assumed that (3) there is a significant three-way interaction of Affective evaluations x Intention x EF performances. Affective evaluations are the strongest predictor of behavior when intention as well as EF performances are both lower compared to higher, and the weakest predictor of behavior when both, intention and EFs are higher compared to lower (Figure 1).

## Materials and Methods

### Participants

Participants were *N*=255 young adults participating at t1 voluntarily or in exchange for course credit. Of these *n*=242 also participated at t2. Due to extreme values in the PA variables at t1 and t2 and problems with data recording in the n-back task (*n*=16), the final sample size consisted of *n*=212 individuals with a mean age of *M*=23.93years (*SD*=2.75). One-hundred-and-sixty of these participants were women (75.5%).

### Procedure

We conducted a prospective study with two points of measurement with 4weeks in between the first (t1) and the second (t2) points of measurement. Participants filled in standardized questionnaires using an online survey tool for quantitative research (Software Unipark QuestBack EFS Survey 10.8 for academic research, Cologne, Germany) and completed standardized reaction time experiments. While the questionnaires were conducted online, the reaction time experiments were lab-based and conducted in the same session, which took place in soundproof cabins and in the presence of an experimenter. The procedure was consistently with the ethical standards of the institutional research committee and with the Declaration of Helsinki. After providing informed consent, sociodemographic and control variables (i.e., age, gender, and past PA behavior) and the independent variable (i.e., affective evaluations) as well as the moderator variables (i.e., intentions and EFs) were assessed at t1. The dependent variable (i.e., PA behavior) was measured 4weeks later at t2.

### Measures

#### Automatic Affective Evaluations

*Automatic* affective evaluations were assessed with a single-target IAT (ST-IAT; Bluemke and Friese, 2008) described in Antoniewicz and Brand (2016). Inquisit 2.0 software (Millisecond Software) was used to control stimulus presentation, log responses and reaction times. Responses were executed on a standard QWERTZ keyboard. Based on a computerized classification task, this test measures the strength of the associations between the target concept PA and attributes of two broad evaluative categories (*good* and *bad*). The attribute words were related to feelings or bodily sensations (see Bluemke et al., 2010) and categorized into good (e.g., “beautiful,” “fantastic,” “magnificent,”) and bad (e.g., “horrible,” “tragic,” “awful,”). The target concept (*physical activity*) was represented by six photographs depicting typical physical activity scenarios without specific affective content (e.g., smiling faces). After a block of 20 practice trials, participants were asked in two blocks to assign attribute words and target (physical activity) pictures to one of two categories which related “physical activity” either to “good” or “bad” (e.g., physical activity + good versus bad or physical activity + bad versus good). Each block consisted of 40 trials. The intertrial interval was 250ms. The between-block difference score (*D*-Score; Greenwald et al., 2003) was calculated (range: −2 to 2) and higher values represented more positive automatic affective evaluations. Please, refer to Antoniewicz and Brand (2016) for further details of this test.

#### Physical Activity Intention

PA Intention was assessed using a standardized scale (Ajzen, 1991; Pfeffer et al., 2020) including 3 items: “I intend to be physically active for at least 30min per day with moderate-to-vigorous intensity,” “I plan to be …,” and “I am determined to be ….” These items had to be answered on 6-point Likert scales from 1 (strongly disagree) to 6 (strongly agree). Internal consistency for the intention scale was calculated at *α*=0.86 in this study, indicating good reliability.

#### Executive Functions

Stimulus presentation as well as RT and correct response measurements in EF tests were performed on a Windows compatible PC. Responses were executed on a standard QWERTZ keyboard. Tests were programmed using the software package Presentation (version 18.1). To assess inhibition a stop-signal task (Verbruggen et al., 2008) and a go/no-go task (Hall et al., 2008) was used. Updating was assessed using an n-back (Smith and Jonides, 1997) and a visual memory task (Salminen et al., 2012). In addition, the data on shifting were obtained by an alternating-runs (Rogers and Monsell, 1995) and the task-cueing paradigm (Sudevan and Taylor, 1987). The methodological details are as follows.

#### Go/No-Go (Inhibition)

For the Go/No-Go task, participants were presented with a series of upper case or lower case letters on the screen. When a LOWER CASE letter is presented, participants are instructed to press the ‘enter’ key as quickly as possible (with the right index finger) while they should refrain from pressing the ‘enter’ key when an UPPER CASE letter is presented. Letters were presented until participants responded, or until 1,600 (the maximal presentation duration) elapsed, and with an inter-trial interval of 500ms. Participants completed a block of 12 practice trials (with equal numbers of upper and lower case letters) after which the experimenter re-emphasized the importance of speed and accuracy, and began the test phase. The test phase consisted of 8 blocks of 60 trials. In half of the blocks lower case letters predominate (‘Go’ phase blocks) and in half of the blocks upper case letters predominate (‘No-Go’ phase blocks). The order of blocks was counterbalanced across participants. RTs were the relevant outcome measure and computed for correct responses only to lower case letters and were computed together for Go and No-Go phase blocks (Hall et al., 2008). Lower values in the outcome measure indicate improved inhibition performance.

#### Stop-Signal Task (Inhibition)

Each trial of this task started with the presentation of a fixation sign (+) for 250ms in the center of the screen. Subsequently, this sign was replaced by the primary-task stimuli “A” and “Z”; fixation sign and stimuli were presented in the center of the screen, in white, on a black background. By default, the response keys were “Y” and “/,” respectively, and participants were instructed to respond as quick and as accurately as possible with their left and right index fingers. The stimuli remained on the screen until participants responded, or until 1,250ms (i.e., the maximal RT) have elapsed. The default inter-stimulus interval was 2,000ms and is independent of RT. Occasionally, a stop-signal (“X,” 75ms) is presented shortly after the stimulus onset in the primary task. On such stop-signal trials, this stop signal is presented after a variable stop-signal delay (SSD). SSD was initially set at 250ms and is adjusted continuously with the staircase tracking procedure: When inhibition was successful, SSD increases by 50ms; when inhibition is unsuccessful, SSD decreases by 50ms. Response registration continues during stop-signal presentation. The experiment consists of two phases: a practice phase of 32 trials and an experimental phase of three blocks of 64 trials (Verbruggen et al., 2008). SSD at the end of the task was the relevant measure. Similar to the Go/No-Go task, lower values in this measure indicated improved inhibition performance in the Stop-signal task.

#### Task-Cueing Paradigm (Shifting)

In this Task-cueing paradigm of the task switching domain, the digits 1 to 9 except 5 were used as stimuli for a magnitude (i.e., < or>than 5) and a parity task (i.e., odd vs. even; Sudevan and Taylor, 1987). Digit stimuli were presented in white color on a black background, in the center of the screen. Colored discs, also presented in the center of the screen and 400ms before the onset of the digit stimulus presentation, were used as task cues. A blue disc indicated the magnitude task, and a red disc indicated the parity task. Responses were given by pressing the Y key (left index finger) and the M key (right index finger). In the magnitude task, participants pressed the left key to indicate smaller than 5 and the right key to indicate larger than 5. In the parity task, participants pressed the left key to indicate even and the right key to indicate odd. The test consisted of an introduction phase (32 trials) and of an experimental phase (5 blocks a 64 trials), including random task presentations. This random presentation resulted in trials of task switches and task repetitions. In the analysis, the task switch effect in form of RTs was relevant: RTs in task switching trials minus RTs in task repetition trials. A reduced task switching effect demonstrated improved shifting performance.

#### Alternating Runs Paradigm (Shifting)

In the Alternating runs paradigm, each trial consisted of the presentation of a character pair including a digit that was either even (2, 4, 6, 8) or odd (3, 5, 7, 9) and a letter that was either a consonant (G, K, M, R) or a vowel (A, E, I, U). One pair at a time was presented in the center of a cell of a 2×2 grid. The first pair of each block appeared in the upper left cell, and the presentation of the following pairs moved clockwise between cells. Each trial lasted until participant’s response, or until 5,000ms had elapsed. The inter-trial interval was 150ms; however, after an erroneous trial it was extended to 1,500ms and also a tone of 30ms in length was presented. Participants were instructed to perform a digit discrimination (i.e., parity) task (even vs. odd) and a letter discrimination task (consonant vs. vowel). They were asked to respond as quickly and as correctly as possible, by pressing a left key with the left index finger for even digits or consonants, and a right key with the right index finger for odd digits or vowels. Altogether 5 blocks were completed. The first two blocks were single-task blocks with 32 trials: one letter discrimination and one digit discrimination block. The last three blocks were mixed blocks with 64 trials, in which both tasks had to be performed so that whenever the stimulus pair appeared in one of the upper cells of the grid, the digit discrimination task had to be performed, and whenever the pair appeared in one of the lower cells of the grid, the participant had to perform the letter discrimination task. Thus, half of the trials in these blocks were trials in which the same task was repeated from one trial to the next, and the other half were switch-trials in which the task switched. Similar to the Task cuing paradigm, the task switch effect in the form of RTs was relevant in this analysis: RTs in task switching trials minus RTs in task repetition trials. Thus, a reduced task switching effect demonstrated improved shifting performance.

#### N-Back Task (Updating)

The participant is visually presented with a sequence of letters. Letters were presented for 2,000ms in the center of the screen with an inter-stimulus interval of 1,000ms. The task consisted of indicating by pressing the enter key when the current stimulus matches the one from two steps earlier in the sequence. Participants performed two blocks with 50 trials each. An increased amount of correct indications of stimulus matches indicated an improved updating performance in this task.

#### Visual Memory Task (Updating)

In this task, the participants performed 1 block with visual stimuli. These stimuli were black bars that appeared one by one in four different locations on the vertical axis of the computer screen. All stimuli were presented for 2,000ms with an inter-stimulus interval of 1,000ms. Each trial included a list of sequentially presented stimuli, and the list lengths were 5, 7, 9, 11, 13, and 15 items (list length was unknown to the participants). In total, ten trials were completed. Immediately following the presentation of each list, participants were asked to report the last 3 item locations of that list in the correct order. So, participants had to constantly update the last three locations during the presentation of the lists. The next list started automatically after three responses. The participants gave their responses by pressing with their right hand the keys “N” for a bar presented in the uppermost part of the screen, “M” for a bar presented slightly above the middle of the screen, “,” for a bar presented slightly below the middle of the screen, and “.” for a bar presented in the lowermost part of the screen. The outcome measure was the number of correctly reported 3-item sequences; an increased number indicated an improved updating performance.

#### Physical Activity Behavior

PA behavior was measured at t1 (as control variable) and t2 (as dependent variable) with four items derived from the IPAQ short-form (Craig et al., 2003) questionnaire. Participants were asked to indicate how many times they performed (a) and (b) moderate physical activities during their spare time during the past 4weeks. Furthermore, participants indicated how long (minutes) these activities were performed on average per occasion. The PA score was calculated by multiplying frequency (during the last 4weeks) with average duration per occasion (minutes) for (a) vigorous and (b) moderate physical activities and subsequently summing up these values. This short questionnaire has acceptable measurement properties with a re-test reliability of 0.80 and a fair to moderate criterion validity (pooled *ρ* =0.30) compared with objective accelerometer data (Craig et al., 2003).

### Statistical Analyses

The program IBM SPSS 23 was used for data screening and data analyses. EF factor scores for inhibition, updating, and shifting were calculated within an exploratory factor analysis inserting the six tests simultaneously based on regression method. To test the hypotheses, a hierarchical multiple regression analysis was conducted. Age, sex, and PA behavior at t1 (i.e., past PA) were included as control variables in the first step. In the second step, implicit associations, intention, and EFs were entered, and in the third step, their two-way interaction terms (we did not expect interaction effects between the EF function domains). The three-way interaction IAT x Intention x EFs were included in step 4. Significant interaction effects were further analyzed by conducting simple slope analyses and slope difference tests (Aiken and West, 1991; Dawson and Richter, 2006; Dawson, 2014). Because of the different scaling, predictor and control variables were standardized (z-transformed) in the case of continuous variables and dummy coded in the case of dichotomous variables prior of calculating the two- und three-way interaction terms. The variance inflation factors for the independent variables in this model were calculated at ≤1.55, indicating no problem with multi-collinearity (tolerance ≥0.64; Menard, 1995). Cook’s distance (*max*=0.20) and leverage values (*max*=0.53) are ≤1 and therefore within the boundary.

## Results

### Descriptive Statistics

Table 1 presents descriptive statistics for and relations among study variables. PA level at t1 for the sample was *M*=211.98min (*SD*=179.89) during the previous week. Intention was significantly correlated with PA at t1 (*r*=0.43) and PA at t2 (*r*=0.32), while PA at t1 and t2 were also significantly associated (*r*=0.48). Further, sex was significantly associated with PA at t1 (*r*=0.15). Men indicated to be more physically active than women. IAT D-Score and EFs did not significantly correlate with PA at t1 or t2.

**Table 1:** Means, standard deviations and correlations of control variables, predictors and the dependent variable of the study (*n*=212).

	1	2	3	4	5	6	7	8	9	10	11	12
Age (1)	–	0.18^**^	−0.05	−0.08	−0.06	0.11	−0.04	0.10	0.06	0.01	−0.14^*^	−0.07
Sex (2)		–	0.15^*^	−0.05	−0.05	0.02	−0.12	0.06	0.04	0.09	−0.09	0.03
PA t1 (3)			–	−0.10	0.43^***^	0.08	−0.06	−0.02	0.02	−0.07	0.04	0.48^***^
IAT (4)				–	0.11	0.05	0.09	−0.00	0.07	0.07	−0.04	0.04
Intention (5)					–	0.06	0.08	−0.12	−0.02	−0.06	0.05	0.32^***^
GoNoGo (6)						–	0.23^**^	−0.26^***^	−0.14^*^	0.09	0.16^*^	0.01
Stop Signal (7)							–	−0.10	0.02	−0.03	−0.03	0.01
N-back (8)								–	0.26^***^	0.04	−0.11	−0.04
Visual Memory (9)									–	−0.08	−0.14^*^	0.07
Alternating-runs (10)										–	0.37^***^	−0.03
Task cueing (11)											–	−0.04
PA t2 (12)												–
*M*	23.93	75.5%^a^	211.98	0.03	3.38	477.26	271.56	0.64	0.41	659.01	130.80	214.81
*SD*	2.75	–	179.89	0.30	1.44	53.45	132.63	0.18	0.24	274.39	93.49	209.29

* *p<0.05;*

** *p<0.01;*

*** *p<0.001;*

a *percentage female. PA, Physical Activity*.

### Hierarchical Regression Analysis

The results of step 1 of the hierarchical multiple regression analysis (Table 2) revealed that age and sex were not associated with PA at t2 (minutes/week), while PA at t1 was, *β*=0.48, *p*<0.001, ∆*R^2^*=0.23, ∆*F*(3,208)=21.09, *p*<0.001.

**Table 2:** Hierarchical multiple regression analyses with IAT, intention, EFs and their interactions as predictors of PA at t2 (controlling for age, sex and PA (t1); *n*=212).

	Step 1			Step 2			Step 3			Step 4		
	B	*R^2^*	*F*	B	*R^2^*	*F*	B	*R^2^*	*F*	B	*R^2^*	*F*
		0.23	21.09^***^		0.26	8.68^***^		0.28	5.11^***^		0.32	5.04^***^
Age	−8.01			−7.69			−5.75			−8.39		
Sex	−15.39			−8.07			−8.93			−2.45		
PA t1	185.39^***^			167.22^***^			169.06^***^			165.65^***^		
IAT				12.71			11.72			3.63		
Intention				26.26^+^			21.47			19.18		
Inhibition				2.16			6.85			−1.19		
Updating				8.66			4.21			7.12		
Shifting				−8.69			−13.77			−7.50		
IAT x Intention							−1.67			−13.13		
IAT x Inhibition							2.24			9.29		
IAT x Updating							−4.70			−3.65		
IAT x Shifting							13.04			4.80		
Intention x Inhibition							18.84			14.33		
Intention x Updating							2.51			4.37		
Intention x Shifting							−32.27^*^			−29.08		
IAT x Int x Inhibition										28.93^*^		
IAT x Int x Updating										−9.31		
IAT x Int x Shifting										−28.06^+^		

*B in respective step; +p<0.10;*

* *p<0.05;*

*** *p<0.001;*

*PA, Physical Activity; Int, Intention; IAT, Implicit Association Test (D-Score)*.

**Figure 2:**
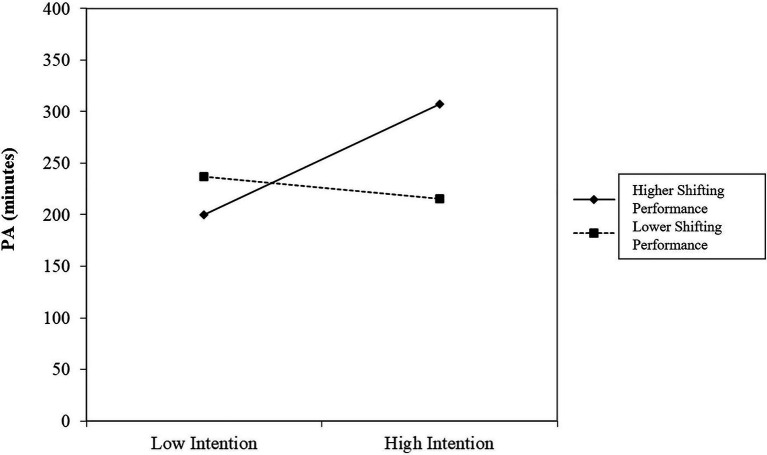
Moderating effect of shifting for the intention–behavior relationship. Note that higher shifting performance is referred to low shifting costs while lower shifting performance is referred to high shifting costs. PA (minutes)=physical activity in minutes at t2.

In step 2, no variable significantly increased the explained variance in PA, ∆*R^2^* =0.02, ∆*F*(5,203)=1.18, *p* =0.32. Also in step 3, no significant increase in explained variance was observed, ∆*R^2^* =0.03, ∆*F*(7,196)=1.03, *p* =0.41. However, the two-way interaction Shifting x Intention became significant, *β* =−0.14, *p* =0.04. Subsequent moderation analyses revealed that the association between intention and PA was stronger, when shifting performance was higher compared to lower (Figure 2). Further, simple slope tests showed that the gradient of the slope was significant for improved shifting abilities, 53.47, *t* =2.58, *p=0*.01, but not for lower shifting abilities, −10.80, *t* =−0.48, *p=0*.63.

**Figure 3:**
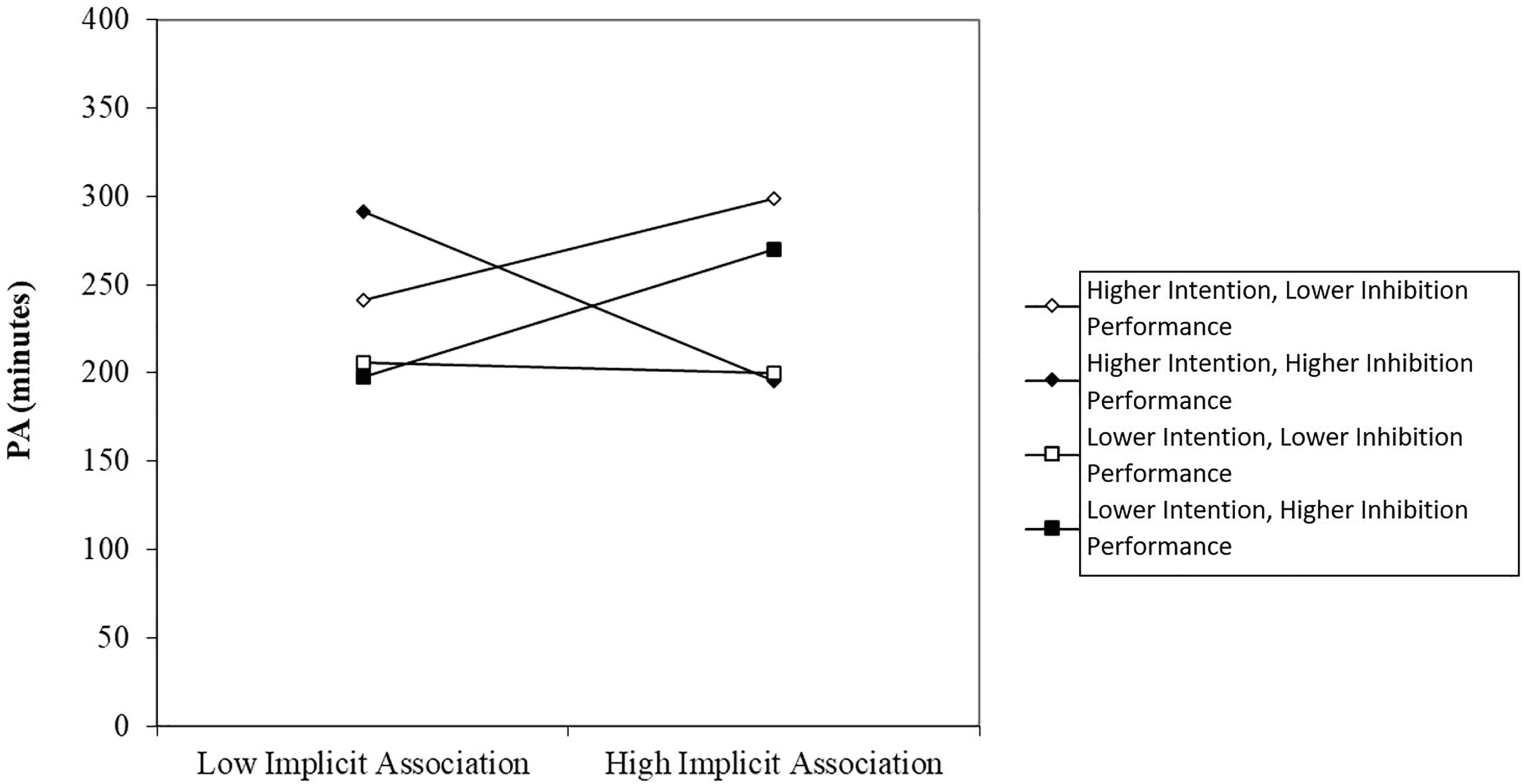
Three-way interaction effect IAT x Intention x Inhibition on PA. Note that higher inhibition performance is referred to lower inhibition costs, while lower inhibition performance is referred to higher inhibition costs. PA (minutes)=physical activity in minutes at t2.

In step 4, the three-way interactions explained a significant amount of variance beyond the previous steps, ∆*R^2^* =0.04, ∆*F*(3,193)=3.62, *p* =0.01. The interaction effect IAT x Intention x Inhibition was significant, *β* =0.16, *p* =0.02 (Figure 3). Moderation analyses revealed that participants with higher intentions and improved inhibition abilities showed a negative association between IAT and PA, while those with lower intentions and improved inhibition abilities showed a positive association between IAT and PA, which was documented in a significant slope difference test between these two groups, −84,11, *t* =−2,06, *p* =0.04. Furthermore, participants with higher intentions and poorer inhibition performance also showed a positive association between affective evaluations and PA and their slope therefore differed marginally significantly from those with higher intentions and improved inhibition abilities, slope difference 76.45, *t* =1.80, *p* =0.07. The other slopes did not differ significantly from each other.

## Discussion

In the present study, we aimed to examine the impact of PA affective evaluations, intentions, and EFs as predicting factors for PA in the framework of dual-process models (e.g., Strobach et al., 2020). Therefore, we assessed automatic affective evaluations (using an ST-IAT), PA intentions, and EFs (Shifting, Updating, Inhibition) as predictors and moderators and tested the direct effects and two-way as well as three-way interactions of these variables on PA within one statistical model. The proposed effects were conducted in a prospective study with two points of measurement with 4weeks in between the first (t1) and the second (t2) points of measurement.

In Hypothesis 1, we assumed that PA affective evaluations, intention, and EFs are positive predictors of PA behaviour. Inconsistent to our hypothesis, the results indicated that none of these variables predicted this behavior. That the IAT D-score was no predictor of PA behavior which is inconsistent to previous studies (e.g., Conroy et al., 2010; Cheval et al., 2015; Hannan et al., 2019) and our hypothesis. However, other studies also failed to show this direct relationship (Padin et al., 2017; Muschalik et al., 2018). This lacking relationship might be explained by the implicit method, with which affective evaluations were assessed. Automatic processes were measured through an IAT while PA behavior was measured with a self-report questionnaire, which are different assessment methods. Furthermore, affective evaluations are a very distal variable of PA behavior, which also might explain the lacking direct relationship between affective evaluations and PA behavior in this study.

The fact that EFs were not directly associated with PA behavior is in contrast to the findings of previous studies (Hall et al., 2008; Chevance et al., 2018), even though other studies only found direct relationships between specific EFs and health behavior (e.g., updating; Allom and Mullan, 2014; Pfeffer and Strobach, 2017). However, these studies substantially differ in how they assessed EFs. Studies often focused on only one domain of EFs (e.g., inhibition) measured by only one task. For example, Hall et al. (2008) solely assessed inhibition with a Go/NoGo-task while Chevance et al. (2017) used the Wisconsin Card Sorting Test, which is a more global task with regard to EFs, and incorporates the shifting domain and the inhibition domain at the same time (Miyake et al., 2000). In this study, we assessed the three EF domains postulated by Miyake et al. (2000) with two different tests each and combining these two tests to a factor score based on a factor analysis. Thereby, we accounted for overlaps between these three domains. Furthermore, in the study of Pfeffer and Strobach (2017) the intention–behavior gap was used as dependent variable, whereas PA behavior was the dependent variable in this study. Therefore, future studies should assess EFs more comprehensively and holistic in order to test systematically which EF domains are most important for PA behavior self-regulation in general and specifically in the context of dual-process theories.

The lack of the postulated relationships might be due to the fact that we controlled for past PA behavior assessed at t1, which is often the strongest predictor of future behavior (Weinstein, 2007). Therefore and not surprisingly, a major amount of the future behaviour might have been explained with the past PA already, leaving not much to explain for the postulated direct relationships. Controlling for past PA, in contrast to no control of such activity, might lead to an underestimation of the contribution of the tested predictors on future behavior (Weinstein, 2007). Furthermore, past behavior can be seen as one aspect of a habit, as cue–response relationships develop through frequent enactment of a behavior in the past. However, we did not measure habit (e.g., automaticity of behavior or context stability) in this study, which prevents us from testing this explanation even though, as already stated above, habits and automatic affective evaluations might share some variance (De Houwer and Moors, 2012; Hagger, 2020).

In Hypothesis 2, we assumed that PA affective evaluations and intention, PA affective evaluations and EFs (Inhibition, Updating, and Shifting) as well as intention and EFs interact in predicting PA behavior. The results of our hierarchical regression analyses revealed that only the interaction of intention and shifting was significant in step 3. Moderation analyses revealed that the association between intention and PA was stronger, when shifting costs were lower (indicating improved shifting performance) compared to higher (indicating impaired shifting performance). Thus, individuals with improved shifting performance and skills more likely transferred their PA intention into actual PA behaviour. This interaction of shifting and intention is consistent with theoretical assumptions. Although not a matter of course, the self-regulatory strategy of means-shifting (Hofmann et al., 2012) may help individuals to flexibly circumvent barriers that may arise (e.g., no time to attend the fitness class) and change plans in terms of activity substitution (e.g., exercising later on the cross-trainer at home) based on a spontaneous decision (Kelly and Updegraff, 2017). The shifting ability seems to be crucial for goal attainment particularly when obstacles occur that impede the initial PA plan. These obstacles can be overcome by selecting and executing alternative means to pursue the same goal. In line with this assumption, Kelly and Updegraff (2017) found that shifting ability predicted higher PA levels through greater means-shifting.

However, the other two-way interactions were not significant, indicating that our hypothesis was only partly supported. As stated above, shifting was examined only rarely in previous studies and there might be substantial overlap between the three EF domains. As we assessed the three domains with two tests each, we analysed the variability of these domains separately and also controlled for task-specific effects, respectively. In detail, when investigating all three EF domains, they are substantially correlated and share a substantial amount of variation (between 0.38 and 0.77, Miyake and Friedman, 2012). That is, the explained variation of each individual EF is reduced by the inclusion of all EFs. In addition, these EFs were investigated on a ‘purer’ latent level with two tests per EF domain. That is, by combining the results of two tests, we also excluded the test-specific impact to predict PA behaviour. Although this strategy reduces the statistical association between the independent EF variables and this behaviour, it improves the conclusions from our study since the exclusion of test-specific predictions decreases the task-impurity problem (Miyake et al., 2000), that other studies suffered from (e.g., Hall et al., 2008).

In Hypothesis 3, a significant three-way interaction of affective evaluations, intentions, and EFs was assumed, and that affective evaluations are the strongest predictor of behavior when intention as well as EFs are both lower compared to higher, and the weakest predictor of behavior when both, intention and EFs are higher compared to lower. Step 4 of our hierarchical regression analysis revealed that the interaction of affective evaluations, intention, and updating as well as of affective evaluations, intention, and shifting were not significant (despite a marginal interaction of the latter combination). However, our data revealed a significant interaction of affective evaluations, intention, and inhibition. Moderation analyses showed that participants with higher intentions and lower inhibition values (improved inhibition abilities) showed a negative association between IAT D-score and PA behavior, while those with lower intentions and lower inhibition values showed a positive association between IAT and PA behaviour. An explanation for this finding could be that individuals who find PA very attractive do not need to employ their inhibition capacity to support their PA but, to the contrary, to engage in activities that are considered as less attractive (e.g., studying for university). This is partly in line with theoretical assumptions and the initial hypothesis. Individuals with negative automatic affective evaluations towards PA are highly physically active, when they have higher intentions and better abilities to inhibit automatic responses such as automatic affective evaluations and the associated approach or avoidance impulses (Strack and Deutsch, 2004; Brand and Ekkekakis, 2018; Strobach et al., 2020).

With regard to dual-process theories it can be concluded that the examined variables in this study seem to be relevant automatic and reflective aspects of PA regulation, which was particularly shown in their interaction effects. The results of our study speak in favour for an interactive effect of automatic and reflective processes rather than an additive pattern with direct effects of each variable (Muschalik et al., 2018). Strong intentions and high abilities to inhibit automatic responses are needed in order to overcome negative affective evaluations of PA. In contrast, strong intentions and better inhibition abilities are not beneficial with regard to PA behavior when the affective evaluation of the behavior is positive. This combination leads to an impaired PA level. Furthermore, there was a tendency that individuals with stronger intentions but poorer inhibition abilities benefited from more positive affective evaluations, compared to those individuals with higher intentions and higher inhibition abilities. To account for PA intention (i.e., PA motivation) as a reflective process within a dual-process perspective seems to be highly relevant, as effective self-regulation needs a goal or standard against which self-regulation can purposefully be deployed as well as sufficient motivation to invest effort into reducing discrepancies between standards and actual behavior. Furthermore, the results of our study are also in line with the assumption that sufficient capacity (in terms of, e.g., EFs) is needed to reduce the discrepancy between the goal and actual behavior in light of obstacles and temptations arising and in order to achieve the goal (Carver and Scheier, 1981; Baumeister and Heatherton, 1996; Hofmann et al., 2012). On the other hand, positive affective evaluations can guide behavior in case that intentions and self-regulation abilities are absent. With regard to our hypotheses, it can generally be concluded that the results partly confirmed the initial assumptions even though some results are not in line with the postulations, which might be explained by the unique and complex combination of variables included in this study.

## Conclusion

Traditional health behavior change models mainly focus on the reflective aspects for the adoption and maintenance of a health behavior and often consider intention as the main determinant of behavior. Automatic processes are hardly included in these models. In contrast, dual-process theories integrate automatic and reflective processes and therefore seem to be more suitable to explain PA. From a practical perspective, as well as fostering more positive affective evaluations towards PA, interventions to strengthen PA intentions, and improve EFs through cognitive and physical trainings, could help to increase PA behavior. Affective evaluations can be changed, for example, by repeatedly pairing PA behavior with positive stimuli (evaluative conditioning) which might shift evaluations in a positive direction (Antoniewicz and Brand, 2016). In addition, several studies have shown that changes in EFs correspond to changes in PA and improvements in EFs predicted higher PA levels (Best et al., 2014; Daly et al., 2014; Allan et al., 2016). Although inhibition cognitive training rarely transfers to other laboratory tasks (Strobach et al., 2014), a recent meta-analysis (Allom et al., 2016) across 19 studies revealed a small to medium overall benefit (*d* =0.38) of this type of training intervention on health behaviors such as reducing the consumption of alcohol (Jones et al., 2018) or high-calorie food (Houben and Jansen, 2011). Although speculative at this point, it might be possible that this type of training also transfers to other health behaviors such as PA.

## Data Availability Statement

The raw data supporting the conclusions of this article will be made available by the authors, without undue reservation.

## Ethics Statement

Ethical review and approval was not required for the study on human participants in accordance with the local legislation and institutional requirements. The patients/participants provided their written informed consent to participate in this study.

## Author Contributions

IP is responsible for the research question and the organization of the study. Furthermore, she was responsible for the statistics and writing the manuscript including theory, methods, results, and discussion. TS was responsible for providing EF tests and respective data management. Furthermore, he substantially contributed to all parts of the manuscript (theory, methods, results, discussion). All authors contributed to the article and approved the submitted version.

## Conflict of Interest

The authors declare that the research was conducted in the absence of any commercial or financial relationships that could be construed as a potential conflict of interest.

## Publisher’s Note

All claims expressed in this article are solely those of the authors and do not necessarily represent those of their affiliated organizations, or those of the publisher, the editors and the reviewers. Any product that may be evaluated in this article, or claim that may be made by its manufacturer, is not guaranteed or endorsed by the publisher.
